# Measuring error in the Demographic Index – steps towards a future population statistics system in England and Wales

**DOI:** 10.23889/ijpds.v9i5.2600

**Published:** 2024-09-18

**Authors:** Rosalind Archer, Peshali Diyasena, Emma Grant

**Affiliations:** 1 Office for National Statistics

## Objective

We are developing methods to measure the quality of the Demographic Index at a National Statistics Institute. The Demographic Index is the foundational dataset for a future, transformed, population statistics system for England and Wales. It clusters records for individuals across many years of health, education, and tax data, and assigns them a unique ID.

Previous research has helped us to conceptualise three types of error in the Demographic Index: clustering error, coverage error, and data measurement error. We are currently working on estimating one type of clustering error, whereby records for two individuals are mistakenly assigned the same ID (“false positive clusters”).

## Approach

Previous work has allowed us to identify variables associated with false positive clusters. We are now working on a stratification method, whereby each ID in the Demographic Index is scored according to how likely it is to contain this error. We plan to measure uncertainty using bootstrapping.

## Results and Conclusions

This work is ongoing, and we hope to obtain results by summer 2024.

## Conclusion

It is vital for us to understand and measure error in the Demographic Index because this is necessary for measuring error in any statistics derived from the Index. And it is crucial that population statistics made by any future system should have measures of error. Assuming that our work shows promise, we will continue by measuring other types of error, and by creating use cases to test how these measures can be fed forward into secondary analyses (e.g. population estimation).

